# Efficacy of Ibuprofen Gargle for Oral Lichen Planus: A Single-Center, Placebo-Controlled, Double-Blind, Randomized Crossover Study Trial

**DOI:** 10.7759/cureus.91242

**Published:** 2025-08-29

**Authors:** Yasumasa Kakei, Yumi Kitahiro, Takeshi Ioroi, Masahiko Kashin, Masaki Kobayashi, Asami Morioka, Kazuhiro Yamamoto, Takumi Hasegawa, Ikuko Yano, Masaya Akashi

**Affiliations:** 1 Oral and Maxillofacial Surgery, Kobe University Graduate School of Medicine, Kobe, JPN; 2 Pharmacy, Kobe University Graduate School of Medicine, Kobe, JPN; 3 Oral and Maxillofacial Surgery, Shin-suma General Hospital, Kobe, JPN; 4 Integrated Clinical and Basic Pharmaceutical Sciences, Okayama University, Okayama, JPN

**Keywords:** crossover study, ibuprofen, mouthwash, oral lichen planus, pain

## Abstract

Purpose: Oral lichen planus (OLP) is a chronic, refractory type of stomatitis characterized by abnormal keratinization and often accompanied by pain. The best treatment for OLP, particularly for pain management, remains unclear. This study focuses on the short-term efficacy of ibuprofen gargle for pain relief in patients with OLP.

Methods: In this crossover study, 24 patients with painful OLP were enrolled. One group received an ibuprofen gargle (0.6%) on days one and three to five and a placebo on day two. The other group received a placebo on day one and ibuprofen on days two to five. The primary outcome was the change in pain level, measured by a Visual Analogue Scale (VAS) before and after gargling on days one and two. Additionally, changes in each domain of the Patient-Reported Oral Mucositis Symptom (PROMS) scale were evaluated from days one to five.

Results: There was no significant difference in the degree of reduction in pain VAS values between the ibuprofen and placebo groups before and five and 15 minutes after use of the gargle. However, the PROMS scale showed a significant reduction in dietary restrictions (p = 0.032) in favor of the ibuprofen gargle compared to baseline.

Conclusion: Ibuprofen gargle may help alleviate dietary restrictions associated with oral intake in patients with OLP who experience pain.

Trial registration: This study was registered with the Japan Registry of Clinical Trials (jRCT) (jRCTs051220009).

## Introduction

Oral lichen planus (OLP) is a chronic inflammatory disease that is resistant to treatment. It is characterized by hyperkeratosis and, histopathologically, by a series of inflammatory reactions. These include serrated changes in the saw-toothed appearance of the rete ridges, thickening of the spinous cell layer, and basal cell damage, as well as severe lymphocytic infiltration in the subepithelial region of the oral mucosa and fluid degeneration at the epithelial-intrinsic layer boundary. Because the cause of OLP is unknown and complete remission is rare, treatment is typically symptomatic and immunomodulatory, especially in severe cases [1]. Clinically, OLP manifests as lacy white patches on the buccal mucosa, which may progress to erythematous lesions with erosions and ulcers. Many patients experience discomfort and pain in the affected areas, negatively impacting their quality of life by hindering proper oral hygiene, which exacerbates inflammation and perpetuates a vicious cycle [2].

Steroid-containing ointments are commonly prescribed for symptomatic treatment of OLP in patients experiencing pain. However, steroids can cause multiple side effects, including secondary candidiasis, nausea, refractory reactions, mucosal atrophy, delayed wound healing, and systemic absorption, all of which can significantly impair quality of life [3]. Other therapeutic options, such as tacrolimus, pimecrolimus, thalidomide, low-level laser therapy, photodynamic therapy, and surgical resection for cases with malignant transformation, have been proposed for steroid-resistant OLP, but none have consistently demonstrated long-term efficacy across all patient populations [4]. As a result, finding an effective treatment with minimal side effects remains a challenge.

Ibuprofen, developed in the 1960s, is a potent inhibitor of prostaglandin synthesis with antipyretic, analgesic, and anti-inflammatory properties [5]. Although systemic use of ibuprofen may cause gastrointestinal and renal side effects due to its inhibition of both cyclooxygenase-1 and -2, numerous reviews and meta-analyses have highlighted its efficacy and relatively low toxicity compared to other nonsteroidal anti-inflammatory drugs (NSAIDs) for adults and children [6,7]. A previous study demonstrated the effectiveness of ibuprofen gargles in treating oral mucositis induced by chemotherapy or chemoradiotherapy [8]. Given the rapid local absorption provided by ibuprofen gargles, this study was performed to investigate their potential effectiveness in treating the erosions and ulcers seen in OLP, which can cause significant pain.

## Materials and methods

Ethical considerations

The study was approved by the Clinical Research Ethics Committee at Kobe University (Approval Reference: C210022, approved on 22 March 2022). It was conducted in accordance with the ethical standards outlined in the Declaration of Helsinki and Japan’s Clinical Trial Act. Additionally, the trial was registered with the Japan Registry of Clinical Trials (jRCT) (Trial ID: jRCTs051220009, registered on 22 April 2022, https://jrct.mhlw.go.jp/latest-detail/jRCTs051220009). Before any study procedures were initiated, all patients provided written informed consent after receiving a full explanation of the study’s purpose and methodology. The consent process was conducted by qualified professionals.

Study design and population

This single-center, randomized, double-blind, placebo-controlled crossover trial was conducted at Kobe University Hospital, following the Consolidated Standards of Reporting Trials (CONSORT) guidelines [9]. Eligible patients were identified based on specific inclusion and exclusion criteria. The first patient enrollment was on June 2, 2022, and the last patient enrollment was on July 11, 2024; the trial ended on July 19, 2024.

Inclusion and exclusion criteria

Inclusion Criteria

Patients eligible for this study must have OLP or OLP-like lesions (OLLs) and experience oral cavity pain averaging 20 mm or more on the Visual Analogue Scale (VAS) over the past seven days from registration. Participants must be currently receiving treatment for their lesions, whether systemic or local, and have been using their current medication at a consistent dose for at least 28 days prior to registration. All patients must be at least 20 years of age at the time of consent acquisition and must have provided documented voluntary consent for participation in this clinical study.

Exclusion Criteria

Patients are excluded from participation if they have peptic ulcer disease, concurrent severe or uncontrolled concomitant medical conditions, or a history of hypersensitivity to any component of ibuprofen gargle. Additionally, those with impaired cardiac function or clinically significant heart disease, aspirin-induced asthma, or who use analgesic drugs at least once weekly for chronic pain are not eligible. The study also excludes patients with dementia, psychiatric symptoms, drug addiction, or alcoholism, as well as pregnant or lactating women. Finally, patients deemed inappropriate for participation by the investigator are excluded from the study.

Diagnosis confirmation

All diagnoses of OLP and OLLs were confirmed by a board-certified pathologist through histopathological examination of biopsy samples.

Intervention

During the crossover period, the patients were randomly assigned in equal numbers to either the ibuprofen-placebo (IP) group or the placebo-ibuprofen (PI) group using a permuted block randomization method, stratified by baseline VAS values (20 to <35 mm or ≥35 mm). To maintain blinding, the block size was not disclosed. A biostatistician generated the randomization sequence, ensuring that all participants, care providers, and outcome assessors remained blinded to group assignments.

In the crossover phase, patients in the IP group received an ibuprofen gargle on day one, a placebo gargle on day two, and ibuprofen gargles from days three to five, administered at least once daily. Patients in the PI group received a placebo gargle on day one, an ibuprofen gargle on day two, and ibuprofen gargles from days three to five, also administered at least once daily. Each patient gargled with approximately 10 mL of the solution, ensuring it contacted the affected area for at least 30 seconds (preferably one minute) before expelling. The patients were instructed not to drink water or rinse their mouths for at least five minutes after gargling, with an interval of at least 15 minutes generally maintained between gargles. These endpoint times were based on a previous study investigating the efficacy of ibuprofen gargles for oral mucositis [8,10]. The maximum daily dosage was one 100-mL bottle of the gargle solution. No new treatments for oral lesions were permitted during the crossover period. Concomitant medications for oral lesions that were in use on the first day of enrollment were allowed to continue, provided their dosage and administration remained unchanged throughout the study. Additionally, patients were instructed not to use concomitant medications from 30 minutes before to 15 minutes after using the study drug.

Ibuprofen gargle preparation

The ibuprofen gargle was prepared by the Department of Pharmacy at Kobe University Hospital. Each 100-mL bottle contained 600 mg of ibuprofen (0.6%; Tokyo Chemical Industry, Tokyo, Japan), along with sodium hydroxide, sodium bicarbonate, and hydrochloric acid for pH adjustment (Fujifilm Wako, Osaka, Japan); glycerin (Fujifilm Wako); and a flavoring agent (Marugo, Saitama, Japan). The placebo gargle was identical in taste and appearance but did not contain ibuprofen.

Random allocation

Patients were randomly assigned to either the IP or PI group in a 1:1 ratio using a permuted block randomization method stratified by the baseline VAS values ranging from 20 to <35 or ≧35mm. The block size was not disclosed to maintain blinding. The randomization sequence was generated by a biostatistician, with blinding maintained for patients, healthcare providers, outcome assessors, and biostatisticians. Randomization was conducted for all patients who provided consent and met the inclusion and exclusion criteria. The block size was not disclosed to maintain allocation concealment. The randomization sequence was generated by an independent biostatistician.

Blinding

This trial was conducted under a double-blind design. The ibuprofen and placebo gargles were identical in appearance, viscosity, and flavor to ensure that participants could not distinguish between them. Randomization assignments were concealed from patients, care providers, investigators, and outcome assessors. Only the independent biostatistician had access to the allocation sequence, and unblinding was not performed during the study period because no serious adverse events occurred.

Data collection and management

Investigators notified the coordinators of patient enrollment via email. The coordinators then reviewed eligibility and issued enrollment confirmations, which included the randomization assignment and enrollment number. Patient data were entered into the Research Electronic Data Capture (REDCap) system, a secure electronic platform for clinical research data management, ensuring confidentiality throughout the trial. The principal investigator verified the completeness and accuracy of the data.

Endpoints

Primary Endpoint

The primary endpoint during the crossover phase was the difference in patients’ pain perception following the use of ibuprofen versus placebo gargles. ΔVAS5 stands for (ΔVAS5 ibuprofen − ΔVAS5 placebo). Pain levels were measured on a continuous VAS [11] from 0 mm (no pain) to 100 mm (worst pain), recorded immediately before and five minutes after gargling on days one and two (ΔVAS5_ibuprofen − ΔVAS5_placebo). This was done to assess the effectiveness of the ibuprofen gargle.

Secondary Endpoints

Among the secondary endpoints, the difference in pain perception after using the ibuprofen and placebo gargles was assessed with a VAS immediately before and 15 minutes after gargling on days one and two (ΔVAS15_ibuprofen − ΔVAS15_placebo) to further evaluate the efficacy of ibuprofen. Additionally, differences in pain perception before and after gargling on days three to five were measured to assess sustained effectiveness. Other secondary endpoints were the onset time and duration of ibuprofen’s effect, the changes in each domain of the Patient-Reported Oral Mucositis Symptom (PROMS) scale after ibuprofen gargle administration, the correlation between the overall daily efficacy and the number of gargle administrations per day from days one to five, and the estimated adverse events.

The PROMS scale evaluates patient-reported symptoms of oral mucositis across 10 domains: mouth pain, difficulty speaking, restriction of speech, difficulty eating hard foods, difficulty eating soft foods, restriction of eating, difficulty drinking, restriction of drinking, difficulty swallowing, and change in taste [12].

Sample size

The target sample size was 24 patients, with 12 assigned to each of the IP and PI groups. A previous study involving patients with chemotherapy- or chemoradiotherapy-induced oral mucositis reported a mean ΔVAS ± standard deviation (SD) for pain relief of −12.8 ± 8.4 mm (n = 7) after three days of using an ibuprofen gargle [8]. In patients with a baseline VAS score of ≥30 mm, the ΔVAS ± SD was −15.6 ± 8.1 mm (n = 5).

In this study, for patients with a baseline VAS score between 20 and 34 mm, the expected ΔVAS5 ± SD for the ibuprofen gargle was 15.0 ± 3.0 mm, while the placebo was expected to show less than half the effect of ibuprofen, estimated at a ΔVAS5 ± SD of 7.5 ± 3.0 mm. For patients with a baseline VAS score of ≥35 mm, the ibuprofen gargle was anticipated to have a ΔVAS5 ± SD of 20.0 ± 10.0 mm, with the placebo estimated at a ΔVAS5 ± SD of 10.0 ± 10.0 mm. Assuming a 2:1 ratio between patients with VAS scores of 20 to 34 mm and those with VAS scores of ≥35 mm, the expected within-subject difference in ΔVAS5 (ΔVAS5_ibuprofen − ΔVAS5_placebo) was calculated as 8.0 mm with an SD of 9.0 mm. The ratio of between- and within-subject variance was estimated to be 0.8. With alpha and beta error rates of 0.05 and 0.10, respectively, the estimated sample size required was nine patients per group. To account for approximately 25% of patients being excluded due to noncompliance or withdrawal, a conservative total of 24 patients was chosen for enrollment.

Statistical analysis

Primary Analysis

During the crossover study period, the mean and SD of ΔVAS5 on days one and two were calculated, along with the 95% confidence interval (CI) for the mean of ΔVAS5. The mean and SD of the within-study ΔVAS5 difference were also computed, and the 95% CI for the mean was established. The treatment effect was assessed by dividing the mean of the within-subject ΔVAS5 difference by two, and the p-value was calculated using Student’s t-test.

Secondary Analysis

The mean and SD of ΔVAS15 on days one and two were calculated, along with the 95% CI for the mean. The mean and SD of the within-study ΔVAS15 difference were also computed, and the 95% CI for the mean was determined. The treatment effect was assessed by dividing the mean of the within-subject ΔVAS15 difference by two, and the p-value was calculated using Student’s t-test.

Summary statistics were generated for the onset time and duration of the study drug’s effect at each time point. Time trend diagrams for each domain of the PROMS scale were created using least-squares means, and 95% confidence limits were calculated for each time point. The mean area under the curve and its 95% CI were also computed.

The full analysis set was used for the primary analysis. Sub-analyses were conducted to account for any imbalances in patient backgrounds, considering covariates. All analyses were performed using R software version 4.2.1 (R Core Team, 2022, R Foundation for Statistical Computing, Vienna, Austria).

## Results

Patient characteristics

**Figure 1:**
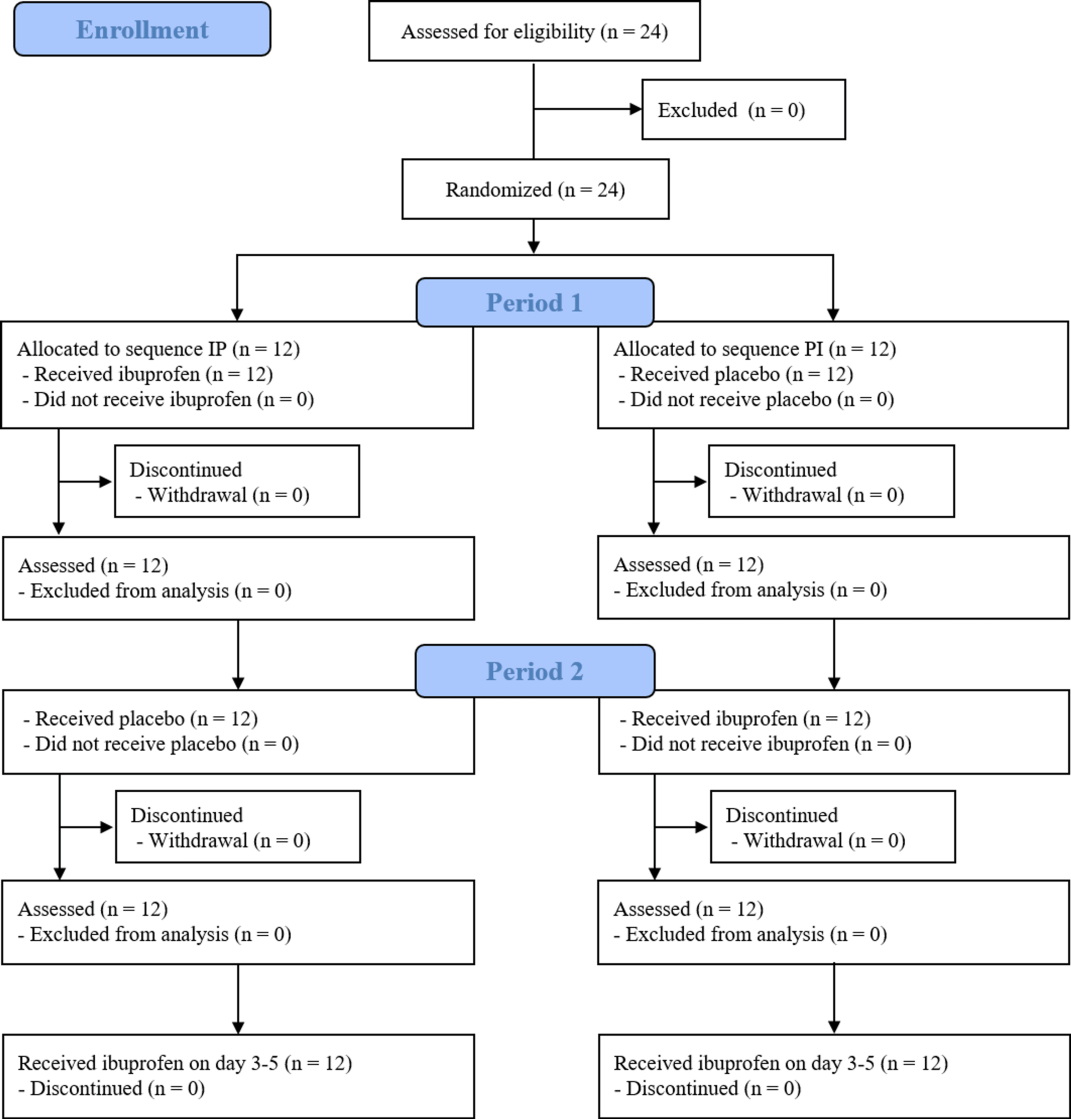
CONSORT flow diagram IP: ibuprofen to placebo; PI: placebo to ibuprofen; CONSORT: Consolidated Standards of Reporting Trials

All patients in this study had OLP. Figure 1 shows the CONSORT flow diagram.

In total, 24 patients were enrolled between June 2, 2022, and July 11, 2024, with 12 patients in each of the IP and PI groups. No patients withdrew their informed consent. The clinical characteristics of all patients are provided in Table 1.

**Table 1:** Patient characteristics 1: Data are expressed as the number of patients (%) or mean ± standard deviation; 2: Data are presented as the average number of lesion sites per patient, with the median number of sites in parentheses. Some patients had multiple lesions at a single site, which is why the total number of buccal mucosa lesions exceeds the total number of patients (12) in each group. IP: ibuprofen to placebo; PI: placebo to ibuprofen; VAS: Visual Analogue Scale; OLP: oral lichen planus

Characteristic	IP group n = 12^1^	PI group n = 12^1^
Age, years	63.3 ± 12.8	63.8 ± 15.6
Sex	-	-
Male	1 (8.3%)	4 (33%)
Female	11 (92%)	8 (67%)
Baseline VAS score, mm	52.3 ± 20.9	53.6 ± 19.1
Duration of OLP, years	4.5 ± 5.2	1.9 ± 1.6
Lesion site^2^	-	-
Number of lesion sites per patient	1.5 (1)	1.75 (2)
Buccal mucosa (total number of right and left)	12	13
Maxillary gingiva (total number of right and left)	3	4
Mandibular gingiva (total number of right and left)	3	3
Tongue (total number of right and left)	0	2
Ongoing treatment for oral lesions	-	-
Azunol gargle liquid	3	3
Dexamethasone ointment	2	3

Efficacy analysis was conducted for all 12 patients in both the IP and PI groups, and safety analysis was performed for all 24 patients across both groups. No protocol violations were identified during periodic monitoring, and the principal investigator did not unblind the study because no serious adverse events occurred that required emergency intervention.

Efficacy outcomes

Table 2 presents the primary endpoint results.

**Table 2:** Results of within-subject difference (VAS5) and treatment effect CI: confidence interval; SD: standard deviation; VAS: Visual Analogue Scale; IP: ibuprofen to placebo; PI: placebo to ibuprofen; ΔVAS5 day one: change in VAS score measured before and five minutes after first use of ibuprofen or placebo gargle on day one; ΔVAS5 day two: change in VAS score measured before and five minutes after first use of ibuprofen or placebo gargle on day two.

Treatment group	Treatment period		Within-subject difference (I–P)
-	ΔVAS_5 Day 1_	ΔVAS_5 Day 2_	-
IP (n = 12)	-	-	-
Mean ± SD	−12.50 ± 17.24	−13.33 ± 21.72	0.83 ± 20.46
95% CI	−23.46 to −1.54	−27.14 to 0.47	−12.16 to 13.83
PI (n = 12)	-	-	-
Mean ± SD	−0.92 ± 19.52	1.67 ± 33.67	2.58 ± 26.84
95% CI	−13.32 to 11.49	−19.73 to 23.06	−14.47 to 19.64
Treatment effect (n = 24)	-	-	-
Mean ± SD	-	-	1.71 ± 23.36
95% CI	-	-	−8.39 to 11.81
t-test for paired samples	-	-	0.729

The within-subject ΔVAS5 scores were 0.83 ± 20.46 mm for the IP group and 2.58 ± 26.84 mm for the PI group. The treatment effect of the ibuprofen gargle was 1.71 mm (95% CI: −8.39 to 11.81; p = 0.729). The carryover and period effects were −26.58 mm (95% CI: −61.64 to 8.47; p = 0.130) and −0.88 mm (95% CI: −10.98 to 9.23; p = 0.859), respectively.

For the ΔVAS15 scores, the within-subject differences were −4.42 ± 13.06 mm for the IP group and −1.50 ± 26.28 mm for the PI group (Table 3). The treatment effect of the ibuprofen gargle was −2.96 mm (95% CI: −11.74 to 5.82; p = 0.492), while the carryover and period effects were −19.08 mm (95% CI: −53.65 to 15.49; p = 0.265) and −1.46 mm (95% CI: −10.24 to 7.32; p = 0.734), respectively.

**Table 3:** Results of within-subject difference (VAS15) and treatment effect CI: confidence interval; SD: standard deviation; VAS: Visual Analogue Scale; IP: ibuprofen to placebo; PI: placebo to ibuprofen; ΔVAS15 day one: change in VAS score measured before and 15 minutes after the first use of ibuprofen or placebo gargle on day one; ΔVAS15 day two: change in VAS score measured before and 15 minutes after first use of ibuprofen or placebo gargle on day two.

Treatment group	Treatment period		Within-subject difference (I–P)
-	ΔVAS_15 Day 1_	ΔVAS_15 Day 2_	-
IP (n = 12)	-	-	-
Mean ± SD	−19.92 ± 23.27	−15.50 ± 21.43	−4.42 ± 13.06
95% CI	−34.70 to −5.13	−27.14 to 0.47	−12.71 to 3.88
PI (n = 12)	-	-	-
Mean ± SD	−7.42 ± 22.63	−8.92 ± 24.18	−1.50 ± 26.28
95% CI	−21.79 to 6.96	−24.28 to 6.45	−18.20 to 15.20
Treatment effect (n = 24)	-	-	-
Mean ± SD	-	-	−2.96 ± 20.35
95% CI	-	-	−11.74 to 5.82
t-test for paired samples	-	-	0.492

**Figure 2:**
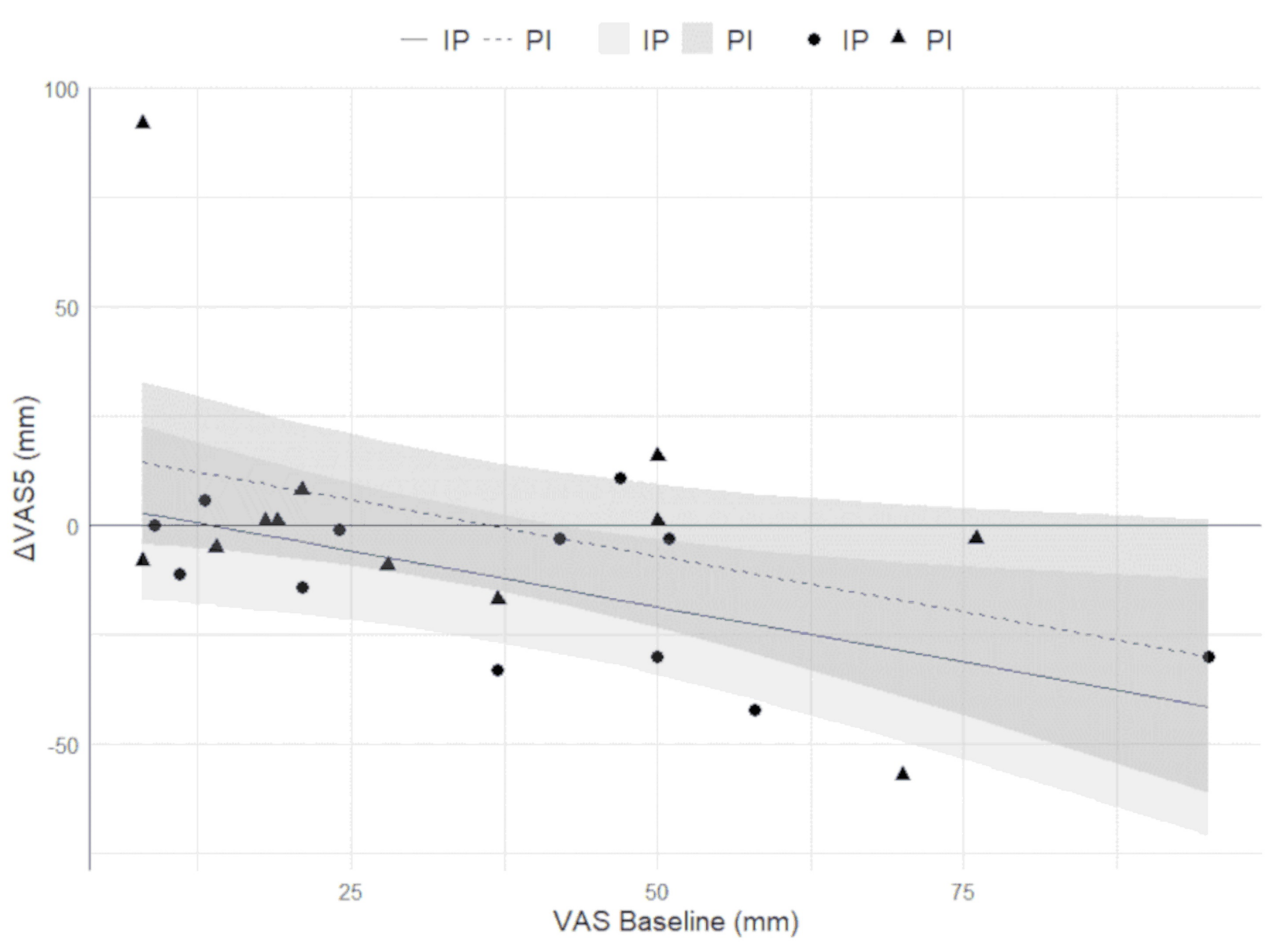
Changes in VAS scores before and after ibuprofen gargle use This figure illustrates the ΔVAS from baseline to five minutes after the first use of the ibuprofen or placebo gargle on days one or two, reflecting changes in pain intensity. The Y-axis represents the ΔVAS (the difference in VAS scores before and after gargle use), while the X-axis shows the baseline VAS scores prior to the first gargle use. The graph presents the regression slopes for two groups: the IP group is represented by circles and a solid line, and the PI group is represented by triangles and a dashed line. Shaded areas indicate the 95% confidence intervals for each group. The regression lines depict the relationship between baseline VAS scores and the changes in VAS scores after gargle use, showing the potential effectiveness of the ibuprofen gargle compared with the placebo. The slopes and confidence intervals were adjusted using an analysis of covariance model, which accounts for baseline differences in VAS scores between the two groups. IP: ibuprofen to placebo; PI: placebo to ibuprofen; VAS: Visual Analogue Scale

Figure 2 illustrates the ΔVAS values from baseline to five minutes after the first use of the ibuprofen gargle on days one or two, indicating a reduction in pain intensity.

The regression lines show the relationship between baseline VAS scores and the changes in VAS scores after gargle use, highlighting the potential efficacy of the ibuprofen gargle compared with the placebo. The slopes and CIs were adjusted using an analysis of covariance model to account for baseline VAS score differences between the groups.

**Figure 3:**
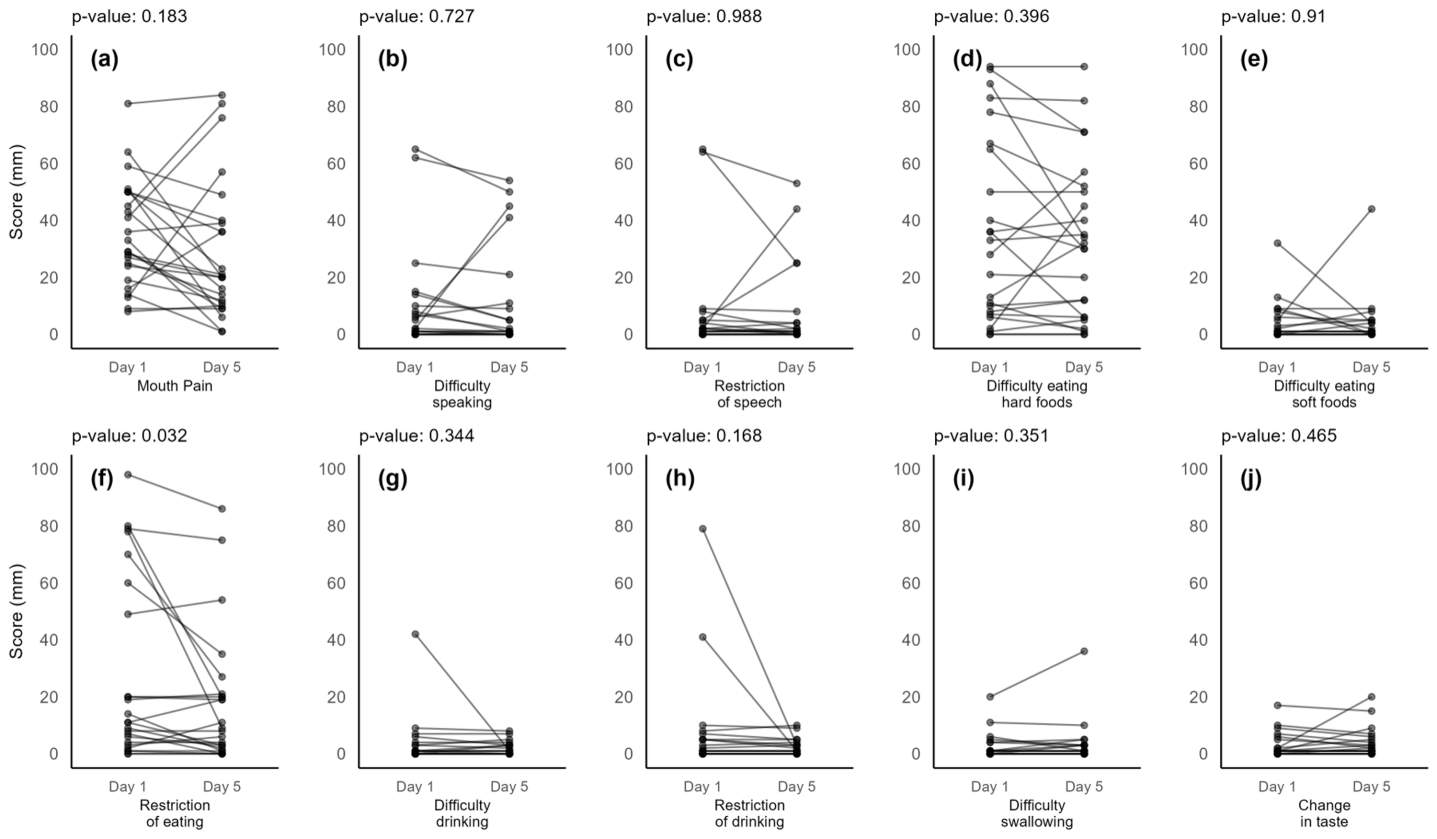
Changes in the 10 domains of the PROMS scale scores between days one and five The 10 subdomains are indicated by (a) to (j). Each plot shows paired data points for individual patients between days one and five, with lines connecting the points to represent changes over time. The Y-axis represents the scores in millimeters on the Visual Analogue Scale, while the X-axis shows the two assessment points: day one and day five for each domain. The p-values from paired t-tests are displayed as subtitles beneath each plot, indicating the statistical significance of score changes over time for each domain. PROMS: Patient-Reported Oral Mucositis Symptom

Figure 3 shows changes in each domain of the PROMS scale from days one to five.

A significant reduction in dietary restrictions was observed on the PROMS scale (p = 0.032).

Safety assessment

No adverse events or clinically significant complications were observed in either group.

## Discussion

This single-center, double-blind, placebo-controlled, crossover study evaluated the efficacy and safety of ibuprofen gargle in reducing daily pain in patients with OLP. Our findings showed that the primary endpoint (the treatment effect of the ibuprofen gargle) was not statistically significant, with a difference of 1.71 mm (95% CI: −8.39 to 11.81; p = 0.729) for VAS5 scores. Similarly, the VAS15 scores showed a non-significant treatment effect of −2.96 mm (95% CI: −11.74 to 5.82; p = 0.492). These results were substantially lower than anticipated in the power calculation, likely reflecting the small sample size. Additionally, a placebo effect may have contributed to the reduction in VAS scores observed in the placebo group. In terms of safety, no adverse events were reported in either the ibuprofen or placebo group. This study aimed to assess the treatment effect while maintaining the standard of care, and our findings provide valuable insights into the potential use of ibuprofen gargle for pain management in OLP.

OLP is a chronic inflammatory disease caused by a T-lymphocyte reaction to basal epithelial cells and is considered a precancerous lesion. It has been reported that 0.07% to 5.80% of cases progress to oral squamous cell carcinoma [13-16], with a recent large-scale meta-analysis suggesting a rate of approximately 1% [17]. Many oral surgeons conduct regular follow-up examinations because of concerns about malignant transformation. The standard treatment involves the topical application of steroids, but long-term use carries a high risk of *Candida* infection [18]. Therefore, a treatment that suppresses inflammation through topical administration without relying on a steroid ointment is desirable.

However, oral administration of NSAIDs is associated with several side effects, making topical NSAIDs a preferable alternative to minimize these risks [19]. Several studies have investigated the effects of topical treatments for OLP-related pain. A small-scale randomized study compared the topical application of morphine to a placebo but found no significant differences in pain relief between the groups [20].

We previously reported that ibuprofen gargles could be an effective local drug delivery system for pain relief after tooth extraction with minimal systemic effects [8]. However, that study involved only ibuprofen-containing treatments without a placebo or control group. The current clinical trial was designed as a placebo-controlled comparative study to evaluate the placebo effect. We assessed the efficacy of ibuprofen gargle versus placebo for pain relief in patients with OLP and OLLs. Additionally, because OLP is associated with erosions, ulcers, and loss of keratinized mucosa, topically administered ibuprofen is expected to be rapidly absorbed into the affected areas.

The primary endpoint of this study was not met because the ΔVAS results varied widely among patients, which did not align with our preliminary power analysis. The pain associated with OLP is chronic and fluctuates daily. In a crossover design, it may be more appropriate to assess pain cumulatively over approximately one week for both placebo and active treatments, rather than through a single evaluation. Additionally, while we set a baseline VAS value of ≥20 mm for this study, a higher baseline VAS value might have provided a clearer analgesic effect because lower baseline values tend to yield smaller effects. Future study designs should aim to reduce the high variability (SD) of VAS scores by incorporating a more focused patient population or ensuring balanced baseline factors, such as VAS values. Furthermore, it may be necessary to implement a longer crossover period with an exchange phase lasting at least one week to better evaluate pain relief.

To minimize the risk of systemic absorption, the ibuprofen gargle solution was held in the mouth for one minute before being expelled. The concentration of ibuprofen used in the study (600 mg ibuprofen/100 mL) was comparable to the recommended maximum daily dose for oral ibuprofen (600 mg). Therefore, any amount of the drug accidentally ingested would likely be below the maximum daily allowance. No severe adverse events were reported in either the ibuprofen or placebo group, indicating that the ibuprofen gargle is acceptable from a safety perspective.

This study had two major limitations. First, the sample size was small. Phase II studies are often based on prior research, but there have been no previous studies on the use of ibuprofen gargling. As a result, when this study was planned, we were unable to accurately estimate the primary endpoint or within-subject VAS scores before and five minutes after the first use of the ibuprofen or placebo gargle on days one and two. Consequently, the results were significantly lower than anticipated in the power calculation. Additionally, the study included patients with diverse background factors, leading to an imbalance in sex and disease duration between the two groups. Although randomization was performed using a substitution block method stratified by baseline VAS scores, sex, and disease duration were not included as stratification factors. Although we attempted to adjust for covariates in the sub-analysis, the small sample size may have limited our ability to draw meaningful conclusions. While no adverse events associated with ibuprofen were observed in this study, suggesting that ibuprofen gargle therapy was well tolerated, it is possible that we overestimated its safety. Larger-scale studies are needed to confirm these findings. Second, this study was conducted over just five days. Participants are allowed to enroll in a six-month continuous trial of the actual drug if they wish to do so [21]. Long-term trial results are necessary to assess both the long-term benefits, such as dietary improvements, and the safety profile of the treatment. In the subsequent long-term extension study, all patients experiencing adverse events or clinically significant conditions will be followed until these events resolve, stabilize, or are deemed no longer clinically significant by the investigator.

## Conclusions

In this randomized, double-blind, placebo-controlled crossover study, the primary pain endpoint was not met; however, ibuprofen gargle was well tolerated and yielded a statistically significant improvement in functional eating (the PROMS “restriction of eating” domain) over the five-day treatment. Taken together with its favorable safety profile and topical mechanism that may limit systemic exposure, these findings support a cautious, symptom-oriented role for ibuprofen gargle in OLP.

Clinically, improving the ability to eat is meaningful for patients with painful OLP, particularly when systemic NSAIDs are undesirable. Future trials should be adequately powered, extend the blinded treatment phase (≥1 week), prespecify patient-centered functional outcomes, and explore baseline pain strata to clarify who benefits most.
